# Yeast GSK-3 kinase regulates astral microtubule function through phosphorylation of the microtubule-stabilizing kinesin Kip2

**DOI:** 10.1242/jcs.166686

**Published:** 2015-11-01

**Authors:** Hauke Drechsler, Ann Na Tan, Dimitris Liakopoulos

**Affiliations:** Biochemistry Centre Heidelberg (BZH), INF 328, Heidelberg 69120, Germany

**Keywords:** GSK-3, Kip2, EB1, CLIP-170, Yeast

## Abstract

The *S. cerevisiae* kinesin Kip2 stabilises astral microtubules (MTs) and facilitates spindle positioning through transport of MT-associated proteins, such as the yeast CLIP-170 homologue Bik1, dynein and the adenomatous-polyposis-coli-related protein Kar9 to the plus ends of astral MTs. Here, we show that Kip2 associates with its processivity factor Bim1, the yeast homologue of the plus-end-tracking protein EB1. This interaction requires an EB1-binding motif in the N-terminal extension of Kip2 and is negatively regulated by phosphorylation through Mck1, the yeast glycogen synthase kinase 3. In addition, Mck1-dependent phosphorylation decreases the intrinsic MT affinity of Kip2. Reduction in Kip2 phosphorylation leads to stabilisation of astral MTs, and accumulation of Kip2, dynein and Kar9 at MT plus ends, whereas loss of Mck1 function leads to defects in spindle positioning. Furthermore, we provide evidence that a subpopulation of Mck1 at the bud-cortex phosphorylates Kip2. We propose that yeast GSK-3 spatially controls astral MT dynamics and the loading of dynein and Kar9 on astral MT plus ends by regulating Kip2 interactions with Bim1 and MTs.

## Abstract (precis)

**Summary:** The yeast GSK-3 kinase controls astral microtubule functions by regulating the interaction of the microtubule-stabilising kinesin Kip2 with microtubules and its processivity factor Bim1/EB1.

## INTRODUCTION

The microtubule (MT) plus end is a site of remarkable versatility: owing to its dynamicity it easily explores cellular space, but it can also mediate and maintain stable interactions with other cytoskeletal factors. Functionality of microtubule plus ends can be modified by a set of specialised proteins termed MT plus-end tracking proteins (+TIPs) that are able to stably associate with the growing end of the MT (Akhmanova and Steinmetz, 2008; Perez et al., 1999). Among +TIPs, MAPRE1 (hereafter referred to as EB1) and cytoplasmic linker protein CLIP-170 play a central role: EB1 is required for the plus-end-tracking of other proteins (Akhmanova and Steinmetz, 2008; Honnappa et al., 2009; Vaughan, 2005), whereas members of the CLIP-170 family are positive regulators of MT growth (Brunner and Nurse, 2000; Komarova et al., 2002; Steinmetz and Akhmanova, 2008).

Yeast homologues of EB1 (Bim1) and CLIP-170 (Bik1) are both involved in spindle positioning during mitosis of *S. cerevisiae.* Spindle positioning in budding yeast ensures that the spindle elongates along the mother-bud axis during anaphase and depends on two redundant pathways, the dynein pathway and the Kar9 pathway. The plus-end-directed kinesin motor Kip2 participates in both pathways by transporting +TIPs to the plus ends of astral microtubules (aMTs). In the Kar9 pathway, Kip2 is required for efficient accumulation of the +TIP Kar9 at aMT plus ends (Maekawa et al., 2003). Similar to adenomatous polyposis coli (APC) – a tumour suppressor that links MTs to actin, Kar9 mediates interactions of aMTs with cortical actin that are required for pre-anaphase spindle positioning and nuclear migration close to the bud (Bienz, 2001; Miller et al., 2000; Miller and Rose, 1998). As part of the dynein pathway, Kip2 transports Bik1 and cytoplasmic dynein from the spindle poles to the plus ends of aMTs (Sheeman et al., 2003; Carvalho et al., 2004; Roberts et al., 2014). Dynein is subsequently offloaded from aMTs and immobilised at the cell cortex, where it pulls on aMTs and facilitates correct positioning of the mitotic spindle in anaphase (Moore et al., 2009).

Besides its role in spindle positioning, Kip2 has an intriguing property in budding yeast: it mediates MT stabilisation (Carvalho et al., 2004; Cottingham and Hoyt, 1997; Huyett et al., 1998). Deletion of *KIP2* results in extremely short aMTs, whereas *KIP2* overexpression leads to cells with abnormally long aMTs. Stabilisation of aMTs by Kip2 seems to be coupled to the transport of Bik1 to aMT plus ends (Carvalho et al., 2004).

Glycogen synthase kinase 3 (GSK-3) is a highly conserved kinase with a key role in signalling during development (Doble and Woodgett, 2003, 2007; Kim et al., 2009; Wu and Pan, 2010), as well as in regulation of MT function and chromosome segregation (Wakefield et al., 2003; Tighe et al., 2007; Buttrick and Wakefield, 2008). In migrating cells and developing neurons, GSK-3 regulates cell polarisation by phosphorylating several +TIPs including APC and CLASP2 (Etienne-Manneville and Hall, 2003; Watanabe et al., 2009). However, the role of GSK-3 in MT regulation within other systems, including yeast cells, is poorly defined.

Here, we show that Kip2 physically interacts with Bim1 through its N-terminal extension, which precedes the kinesin motor domain. This extension is heavily phosphorylated by the yeast GSK-3 kinase homologue Mck1 in a cell-cycle dependent manner, and probably requires a priming phosphorylation by the LATS-related kinase Dbf2. We provide evidence that the N-terminal extension is a regulatory hot spot because phosphorylation not only interferes with Bim1 binding, but also reduces the MT affinity of Kip2. We propose that Mck1 and, possibly, Dbf2 control spindle positioning through spatial regulation of aMT dynamics, and the deposition of dynein and Kar9 at aMT plus ends through phosphorylation of the kinesin Kip2.

## RESULTS

### Kip2 is phosphorylated by budding yeast GSK-3/Mck1

**Fig. 1.**
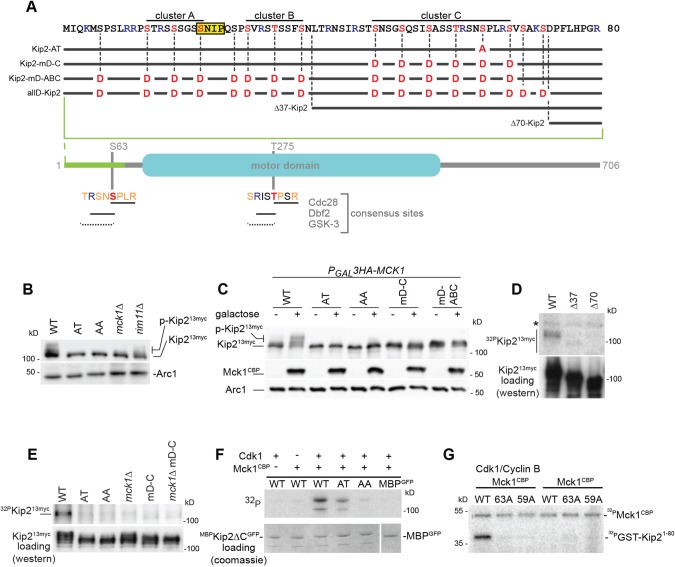
**Mck1 phosphorylates Kip2 *in vivo* and *in vitro*.** (A) Details of Kip2 variants described in this paper and abbreviations used in labels, as well as graphical representation of Kip2. Positions of the GSK-3, Cdc28 and Dbf2 consensus sites, residues S63 and T275 are shown in red. The SxIP Bim1/EB1-binding motif is marked in yellow. (B) Comparison of phosphorylation and expression levels between unphosphorylatable Kip2^13myc^ variants and Kip2^13myc^ in *mck1*Δ and *rim11*Δ cells. Phosphorylation of Kip2^13myc^ is reduced upon introduction of S63A mutation, in *mck1*Δ cells and marginally in *rim11*Δ cells. Shown is a western blot against the Myc epitope in whole-cell extracts. Variant labelling see (A), AA corresponds to Kip2^13myc^-S63A T275A. Arc1 serves as loading control. (C) Depletion of Mck1 leads to decrease in Kip2 phosphorylation. Overexpression of Mck1 does not increase phosphorylation of hypo-phosphorylated Kip2^13myc^ variants mutated in the GSK-3 consensus site(s). ^HA^Mck1 expression from the *GAL1-10* promoter *(P_GAL_)* was induced by addition of galactose (+), depletion (−) by addition of glucose to the medium. Western blot shows Kip2^13myc^ phosphorylation and ^HA^Mck1 expression levels. Arc1 serves as loading control. (D,E) *In vivo* ^32^P-phosphate labelling of Kip2^13myc^ variants and Kip2^13myc^ in different kinase mutants. Kip2 loading: western blot of the Kip2^13myc^ variants after immunoprecipitation from cells labelled with ^32^P-phosphate, the same blot was used for the autoradiography shown. (F) Mck1 phosphorylates Kip2 *in vitro*. ^MBP^Kip2ΔC^GFP^ and displayed variants were pre-incubated with Cdk1 and cyclin B and/or ATP followed by incubation with Mck1^TAP^ and γ^32^P-ATP as indicated. Autoradiography of the subsequent SDS-PAGE is shown. Mck1^TAP^ phosphorylates Kip2 only after pre-incubation with Cdk1 and phosphorylation is abolished after introduction of the S63A and S275A mutations. (G) Sequential phosphorylation of Kip2 N-terminus by Mck1. A fusion of the N-terminal 80 aa of Kip2 to GST (GST-Kip2^1-80^) is phosphorylated only after pre-incubation with Cdk1. Mutation of S63 but also of the upstream T59 abrogated phosphorylation.

Mitotic Cdc28 (the budding yeast Cdk1) phosphorylates Kip2 *in vitro* (Ubersax et al., 2003). In line with this, we identified two potential phosphorylation sites in Kip2 – residues S63 and T275 – that fit the Cdc28 consensus [S/T]PxR sequence, in which x represents any amino acid (aa) (Fig. 1A). Indeed, in western blot analysis of cell extracts, Kip2 C-terminally tagged with 13 Myc epitopes (Kip2^13myc^) displayed a complex migration pattern that collapsed after treatment with alkaline phosphatase (Fig. S1A). This suggested that part of Kip2 is present within cells as a number of phosphoisoforms that display different electrophoretic mobilities on SDS PAGE. Furthermore, replacing S at position 63 with A (Kip2-AT^13myc^) largely abrogated Kip2 phosphorylation (Fig. 1B, Fig. S1A). The single T275A replacement did not show any significant effect *in vivo* (Fig. S1A), whereas combination of S63A and T275A mutations (Kip2-AA^13myc^) displayed similar reductions when compared with Kip2-AT^13myc^ (Fig. 1B, Fig. S1A). We next tested whether Cdc28 phosphorylates Kip2 *in vivo* by inhibiting Cdc28 over time using the *cdc28-as1* strain (Ubersax et al., 2003). In this experiment, the bona fide Cdc28 substrate Kar9 served as an internal positive control (Liakopoulos et al., 2003). Whereas phosphorylation of Kar9^TAP^ decreased rapidly within 20 min after Cdc28 inhibition, Kip2^13myc^ phosphorylation remained fairly stable over 50 min (Fig. S1B). In addition, pairwise deletion of the yeast cyclins Clb1–6, which are essential for Cdc28 activity did not significantly decrease Kip2 phosphorylation (Fig. S1C). Hence, we conclude that Cdc28 is not the main kinase involved in Kip2 phosphorylation.

We took a systematic approach to identify the kinase mainly responsible for Kip2 phosphorylation. We did not detect any reduction in Kip2^13myc^ phosphorylation in cells with mutations in the yeast Polo kinase (*cdc5-2*; Charles et al., 1998), the mitotic exit network kinase Cdc15 (*cdc15-1*; Shirayama et al., 1998) or the yeast kinase Aurora (*ipl1-321*; Biggins et al., 1999; data not shown). Next, we examined Kip2 phosphorylation in 60 strains each of which was deficient for a non-essential kinase. We observed the most prominent reduction in Kip2 phosphorylation in cells deleted for *MCK1*, a kinase that encodes one of four homologues of the GSK-3 kinase in budding yeast (Fig. 1B, Fig. S1D; the other three GSK-3 homologues are Rim11, Mrk1 and Ygk3). Phosphorylation of Kip2 in *rim11*Δ cells is only marginally reduced (Fig. 1B), whereas deletion of Mrk1 or Ygk3 had no significant negative effect on Kip2 phosphorylation levels (Fig. S1D). Moreover, phosphorylation of Kip2 *in vivo* decreased upon downregulation of *MCK1* (expressed under the regulatable *P_GAL1-10_* promoter) and hyperphosphorylation was restored upon overexpression of the kinase – the latter effect was absent upon introduction of the S63A mutation (Fig. 1C). Thus, we conclude that Mck1 is the main kinase responsible for the S63-dependent Kip2 phosphorylation.

### Mck1 phosphorylates the unstructured N-terminus of Kip2

We next searched for regions of Kip2 that are targeted by Mck1. GSK-3 is known to sequentially phosphorylate multiple residues within a continuous S/TxxxS/T pattern upstream of an initial phosphorylation site that is often (but not always) phosphorylated by a priming kinase (Roach, 1991). Such a pattern extends throughout the entire S/T-rich N-terminal region of Kip2 (32 S and T residues within the first 80 aa) and is predicted to be heavily phosphorylated by GSK-3 at three S/T clusters according to several algorithms (clusters A, B and C; Fig. 1A; for site prediction see Materials and Methods). Furthermore, T275 is also part of a GSK-3 consensus site. Thus, mutation of S63 and T275 disrupts not only the Cdc28 but also the GSK-3 consensus site and may abrogate phosphorylation either by inhibiting priming phosphorylation or by stopping the sequential phosphorylation cascade. In line with this, overexpression of *MCK1* failed to induce hyperphosphorylation of Kip2^13myc^ variants with mutations of S63 or in the N-terminal GSK-3 clusters (Fig. 1C). Since a single aa replacement at S63 has the same effect as replacing the S and T residues in the entire N-terminal clusters with D residues, we conclude that S63 is, indeed, crucial for Mck1-dependent Kip2 phosphorylation.

To investigate differences in phosphorylation of the different N-terminal variants of Kip2 *in vitro* (Fig. 1A), we labelled living cells with ^32^P-orthophosphate and examined phosphorylation by autoradiography after immunoprecipitation (Fig. 1D,E). Consistent with our previous results, phosphorylation of Kip2^13myc^ in *mck1*Δ cells was severely reduced, as was that of Kip2-AT^13myc^ (and, to a similar extent, that of Kip2-AA^13myc^). Deletion of the clusters A and B (Δ37-Kip2^13myc^) severely reduced phosphorylation, whereas Kip2 deleted for the whole N-terminus before aa residue 70 (Δ70-Kip2^13myc^) did not exhibit any detectable phosphorylation (Fig. 1D), even when considering that Δ70-Kip2^13myc^ protein levels were clearly lower compared to wild-type Kip2. These data suggest that the S/T-rich terminus of Kip2 bears the main phosphorylation sites and that phosphorylation extends through all three clusters of the GSK-3 consensus site.

We next tested phosphorylation of Kip2 by Mck1 *in vitro*. We, thus, purified from bacteria an MBP-fusion of Kip2 lacking the C-terminal tail (Kip2 1–560) followed by GFP (^MBP^Kip2ΔC^GFP^; this fusion was more stable than full-length Kip2 isolated from yeast and from insect cells) and Mck1^TAP^ from yeast cells. In line with the requirement for a priming-phosphorylation by GSK-3 kinases, Mck1^TAP^ alone was not able to phosphorylate ^MBP^Kip2ΔC (Fig. 1F). However, pre-incubation with recombinant Cdk1 and cyclin B allowed ^MBP^Kip2ΔC to become efficiently phosphorylated after Mck1^TAP^ addition. Introducing a S63A mutation reduced phosphorylation, whereas a double S63A T275A mutation nearly abrogated *in vitro* phosphorylation. These results suggested that priming phosphorylation of S63 by using Cdk11 and cyclin B can support sequential phosphorylation by Mck1 of sites further upstream. Accordingly, mutation of T59 to an unphosphorylatable residue should prevent phosphorylation of the upstream cluster and phenocopy of the S63A mutation. To test this, we constructed ^GST^Kip2^1–80^ fusion proteins bearing the S63A or T59A mutation and examined their phosphorylation *in vitro*. As predicted, both constructs were only poorly phosphorylated compared to the wild-type control (Fig. 1G). Taken together, our results suggest that Mck1 sequentially phosphorylates Kip2 at the GSK-3 clusters within its N-terminus *in vivo* and *in vitro.* It is also likely that the GSK-3 consensus site that comprises T275 is phosphorylated with T275 acting as a priming site. However, we cannot exclude that T275 acts as a priming site only in the presence of the S63A mutation.

### Dbf2 might act as priming kinase for Mck1

Although our *in vitro* experiments suggest that Cdc28 is a priming kinase for Mck1, inhibition of Cdc28 did not affect Kip2 phosphorylation *in vivo* and deactivation of Cdc28 did not decrease Kip2 phosphorylation in absence of Mck1 (Fig. S1B,C,D). Furthermore, S63 is part of cluster C but is not the most downstream Ser within that cluster. We, therefore, reasoned that S69 or S72 might act as a priming site *in vivo*.

**Fig. 2.**
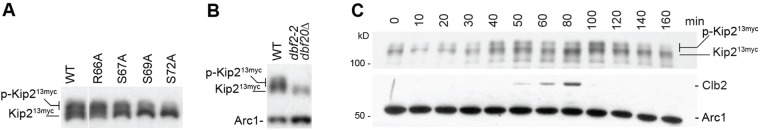
**Dbf2 and additional kinases act on Kip2 as a priming kinases for GSK-3.** (A) Amino acids at positions S69 and S72, downstream of S63 act presumably as phosphorylation priming sites for Mck1. Western blots of the indicated Kip2^13myc^ variants. Kip2^13myc^ phosphorylation is reduced upon mutation of the indicated sites. (B) Kip2^13myc^ phosphorylation is clearly reduced in *dbf2-2 dbf20*Δ mutants. (C) Kip2 phosphorylation is cell-cycle regulated and increases in anaphase (western blot against Kip2^13myc^ of cell extracts from cells synchronized with α-factor for 3 h and released in fresh medium lacking the pheromone, at 30°C).

Indeed, we observed a clear reduction of Kip2^13myc^ phosphorylation upon introduction of S69A and the highest reduction upon introduction of the S72A mutation, suggesting that these sites control phosphorylation of the upstream cluster *in vivo* (Fig. 2A). A consensus site for protein kinase C comprises S69, whereas the aa sites 69–75 are also predicted CKI sites (ELM; http:/elm.eu.org/). However, we did not observe a significant reduction in Kip2^13myc^ phosphorylation in yeast *stt1-1* (PKC) or *yck1*Δ *yck2-2^ts^* (CKI) mutants (Fig. S1E). Therefore, the (priming) kinases that phosphorylate these sites remain unknown.

However, during the analysis of the yeast-kinase deletion strains, we observed that Kip2 phosphorylation is diminished in *dbf20*Δ-null mutants as well as in *dbf2*Δ strains (Fig. S1F). Dbf20 is a close homologue of the LATS-related Dbf2 kinase in yeast. Dbf2 kinase is a part of the mitotic exit network that is required in metaphase for correct spindle orientation (Hotz et al., 2012) and is further activated following anaphase to promote exit from mitosis (Bardin and Amon, 2001). Upon analysis of the *dbf2-2 dbf20*Δ double mutant, we observed that phosphorylation of Kip2^13myc^ was, indeed, clearly reduced (Fig. 2B). Furthermore, we examined phosphorylation of Kip2 during the cell cycle (Fig. 2C, Fig. S1G). Phosphorylation levels of Kip2^13myc^ were lowest during metaphase, followed by increase in Kip2^13myc^ phosphorylation immediately after. Therefore, the pattern of Kip2 phosphorylation fits well with the timing of principle Dbf2 activation. Although some phosphorylation was still evident, the increase of phosphorylated Kip2^13myc^ upon anaphase entry was less pronounced in the *dbf2-2 dbf20*Δ mutant (and the *mck1*Δ mutant) when compared to wild type (Fig. S1G), in agreement with the hypothesis that Dbf2 acts on Kip2 as a priming kinase for Mck1.

Upon closer inspection of the Kip2 sequence, we noticed that residues S13, S63 and S69 within the N-terminal clusters are preceded by an R and, thus, match the preferred sequence for the Dbf2 kinases [RxxS/T] (Mah et al., 2005; Fig. 1A). Furthermore, T14, S18 and S33 are part of such consensus sites although not part of the GSK-3 [SxxxS] pattern. Intriguingly, neither an R60A nor an R66A single mutation (Fig. S1H and Fig. 2A, respectively) caused significant difference in the Kip2^13myc^ phosphorylation pattern. We refrained from mutating more R residues in the N-terminal Dbf2 consensus sites because this would have drastically changed the pI of this domain and might have perturbed Kip2-MT interactions (see below). Our interpretation of these data so far is that priming for Mck1 phosphorylation is mediated by Dbf2 and/or Dbf20 within the Kip2 N-terminus, and possibly by additional kinases that act at S69 and S72.

### Regulation of MT stability through phosphorylation of Kip2

**Fig. 3.**
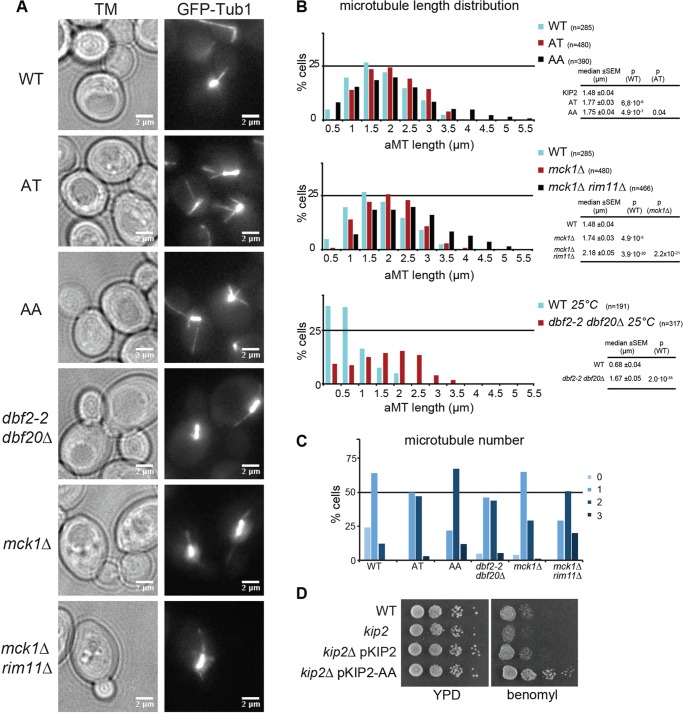
**Mck1 and Dbf2 regulate aMT stability through Kip2 phosphorylation.** (A) Lack of Mck1- and Dbf2-dependent Kip2 phosphorylation stabilises aMTs. Images of MTs from *kip2*Δ cells expressing unphosphorylated Kip2 variants and from depicted kinase mutants. The MTs shown here do not represent the mean MT length but are characteristic for each strain. (B) Length distribution and corresponding statistics for bud aMTs in G2/M-phase cells of depicted strains. Number of counted aMTs are shown in brackets, *P* , statistical significance of the difference between the distribution of the strain on the left of the graph and the MT distribution of the strain denoted in brackets. Imaging of all strains was performed in the same experiment. See Fig. S2B for additional aMT length measurements. (C) The number of aMTs is increased upon a reduction in Kip2 phosphorylation. The number of aMTs growing from either spindle pole was counted; *n*>100 cells for each strain. (D) *kip2*Δ cells show growth sensitivity, and Kip2-AA-expressing cells resistance to the MT-destabilising drug Benomyl. See Fig. S2A for images of Kip2-SA and -SE (T275E mutation) variants.

GSK-3 is an important regulator of MT function in mammalian cells (Buttrick and Wakefield, 2008), but its role in MT regulation in budding yeast is unknown. Kip2 localises exclusively on aMTs and has been reported to promote aMT stability by transporting Bik1 to aMTs plus ends (Carvalho et al., 2004). This prompted us to examine the aMT phenotypes of hypo-phosphorylated Kip2 variants that bear mutations within the GSK-3 consensus site in cells at G2/M phase. For this, we created *kip2*Δ cells that genomically express different Kip2 variants under the control of the native *KIP2* promoter. Interestingly, cells that express Kip2 variants that are defective in Mck1 phosphorylation (Kip2-AT) displayed an increased number as well as abnormally long astral MTs (Fig. 3A,B,C). This phenotype is exacerbated in Kip2-AA-expressing cells, in which the numbers of long aMTs were increased. In addition, these cells were growth resistant to the MT-destabilising drug Benomyl (Fig. 3D). Cells that express Kip2 with point mutations at T275 (S→A or S→E) within the GSK-3 consensus site alone did not display a significant aMT phenotype (Fig. S2A). Consistently, reduction of Kip2 phosphorylation through deletion of *MCK1* also results in aMT stabilisation and increase in aMT number (Fig. 3A,B,C), whereas these aMT phenotypes are even more pronounced in *mck1*Δ *rim11*Δ mutant cells, but not after combining the *mck1*Δ deletion with *mrk1*Δ or *ygk3*Δ (Fig. S2B). Importantly, we also observed the same aMT phenotypes in *dbf2-2 dbf20*Δ cells in all phases of the cell cycle (Fig. 3A,B,C), consistent with the role of Dbf2 as a priming kinase for Mck1. In conclusion, these experiments show that expression of the hypophosphorylated form of Kip2 stabilizes MTs.

### Phosphorylation inhibits the binding of Kip2 to MTs

**Fig. 4.**
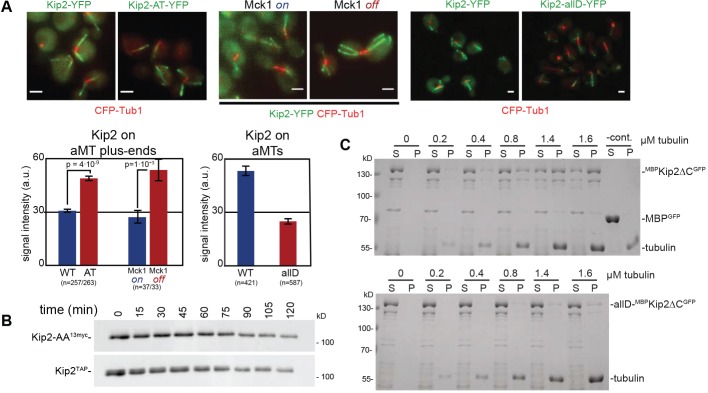
**Phosphorylation regulates association of Kip2 with MTs.** (A) Mck1-dependent phosphorylation regulates Kip2 load on aMTs. Fluorescence intensity of Kip2–YFP, Kip2-AT–YFP and allD-Kip2–YFP in wild-type cells and of Kip2–YFP in ^HA^Mck1-overexpressing or Mck1-depleted cells. Examples of quantified images are given above each graph. Numbers of counted aMTs in brackets, arbitrary intensity units ±s.e.m. are shown. Scale bars: 3 µm. (B) Cycloheximide chase of plasmid-borne Kip2^13myc^AA under control of the *KIP2* promoter in cells expressing Kip2^TAP^ from the *KIP2* locus as an internal control. Kip2 phosphoisoforms were not resolved here in order to facilitate comparison between different Kip2 variants. (C) A variant of Kip2 with a phosphorylation-mimicking N-terminus (allD-^MBP^Kip2ΔC^GFP^) binds less efficiently to MTs *in vitro*, compared to the unchanged variant. Shown are Coomassie-Blue-stained gels of microtubule sedimentation assays, after incubation of *in vitro* polymerised, taxol-stabilized MTs with recombinant and ^MBP^Kip2ΔC^GFP^ or allD-^MBP^Kip2ΔC^GFP^. S, supernatant; P, pellet. Refer also to Fig. S2D.

We next set out to elucidate the molecular mechanism through which Mck1-dependent Kip2 phosphorylation might affect aMT stability. We first reasoned that phosphorylation simply might control the stability of Kip2. In line with this idea, cells expressing a Kip2-AT–YFP variant display a significantly higher plus-end accumulation at aMTs when compared to cells expressing wild-type Kip2–YFP (Fig. 4A). This effect was not specific only for the plus ends but is, rather, a result of the overall increased amounts of Kip2-AT–YFP on aMTs. Consistently, depleting cells of Mck1 also results in increased aMT- and plus-end accumulation of wild-type Kip2–YFP, whereas overexpression of Mck1 had the opposite effect (Fig. 4A). We, therefore, set up a cycloheximide-chase experiment to follow degradation kinetics of different Kip2 variants, and co-expressed Kip2^13myc^-AA and wild-type Kip2^TAP^ as an internal control (Fig. 4B). Both proteins, however, displayed indistinguishable turnover kinetics, suggesting that Mck1-dependent Kip2 phosphorylation does not regulate Kip2 stability and that the increased plus-end accumulation of the hypo-phosphorylated Kip2 must have a different cause.

Increase in affinity for MTs or in processivity of hypo-phosphorylated Kip2 isoforms might also account for increased accumulation at aMT plus ends. In the first case, more Kip2 would be loaded onto aMTs, in the second case loaded Kip2 would be more likely to reach the plus end. We first addressed the possibility that phosphorylation of the Kip2 N-terminus increases the MT affinity of the motor. This unstructured, S/T-rich region is highly basic (with pI=13) and is reminiscent of the Ndc80 N-terminal extension that contributes to binding of Ndc80 to MTs (Ciferri et al., 2008). Interestingly, introducing negative charges by phosphorylation of the N-terminal extension in Ndc80 weakens its interaction with the MT lattice (Ciferri et al., 2008). We, therefore, asked whether phosphorylation of the N-terminal sequence of Kip2 has an analogous effect on MT binding. Recombinant ^GFP^Kip2 purified from insect cells is hyperphosphorylated in an S63-dependent manner in western blots – similar to that from yeast cells – and we verified by using mass-spectrometry that S63 is, indeed, phosphorylated (Fig. S2C,D; see also Roberts et al., 2014). We, thus, asked which forms of purified ^GFP^Kip2 interact stronger with MTs. In MT- sedimentation assays, the affinity of the hyperphosphorylated ^GFP^Kip2 isoforms was reduced compared to hypo-phosphorylated ^GFP^Kip2 (Fig. S2D). Furthermore, to mimic a constitutively phosphorylated Kip2, we mutated all S or T residues to D within the N-terminal cluster of our ^MBP^Kip2ΔC^GFP^ construct (allD-^MBP^Kip2ΔC^GFP^) and repeated the co-sedimentation experiments. Again, wild-type ^MBP^Kip2ΔC^GFP^ co-sedimented with increasing tubulin concentrations, the majority of allD-^MBP^Kip2ΔC^GFP^ remained in the supernatant (Fig. 4C). Consistent with these findings, introduction of the same mutations to the Kip2–YFP construct (allD-Kip2–YFP) reduced Kip2 MT load by ∼50% *in vivo* (Fig. 4A). Therefore, we propose that Mck1 controls aMT stability by phosphorylating the N-terminus of Kip2 and by regulating the affinity of Kip2 for MTs.

### Mck1 controls interaction of Kip2 with the yeast EB1

**Fig. 5.**
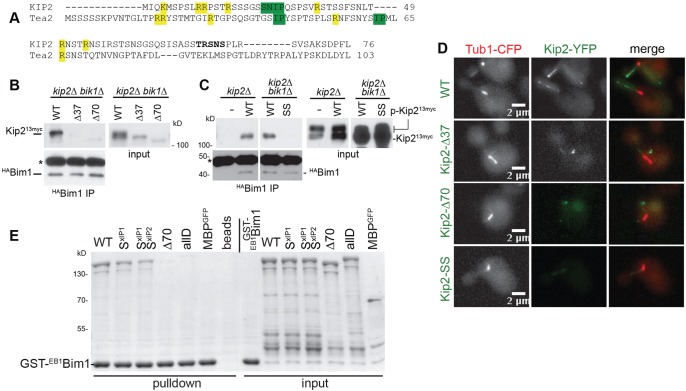
**Kip2 interacts with Bim1 over its N-terminal extension in a phosphorylation-dependent manner.** (A) Alignment of the N-terminal extensions of Kip2 and fission yeast Tea2. The proposed EB1-interacting motifs are shown in green, positively charged residues in yellow. The GSK-3 consensus site comprising S63 is shown in bold. (B,C) Bim1 interacts with the EB1-binding motif at the Kip2 N-terminus, independently of Bik1. Immunoprecipitations of ^HA^Bim1 from *kip2*Δ *or kip2*Δ *bik1*Δ cells expressing depicted plasmid-borne Kip2^13myc^ variants. Note that only non- (or hypo)-phosphorylated Kip2 isoforms co-precipitate with ^HA^Bim1 (seen in C). *P_GAL_3HA-BIM1* expression was induced for 3 h prior to immunoprecipitation. Immunoprecipitated proteins (IP) and extracts after the immunoprecipitation (input) were probed in western blots with anti-HA and anti-Myc antibodies. Asterisk indicates antibody heavy chain. SS refers to the Kip2^13myc^ variant with the SNIP motif mutated to SNSS. (D) Localisation of the Kip2–YFP variants which show decreased interaction with Bim1 (C and D). Representative images of cells expressing CFP–Tub1 and integrated Kip2–YFP variants from the *KIP2* promoter as the sole Kip2 source. (E) The two EB1-binding motifs are required for Kip2–Bim1 interaction *in vitro*. Shown are coomassie stained gels INPUT: amounts of the recombinant proteins used in the pulldown assay, the EB1 domain of Bim1 (^EB1^Bim1) as a GST-fusion was bound to beads (bait). S^xIP1^ and S^xIP2^ denote the recombinant Kip2 variants with respective mutations (SNIP to SNNN) in the two EB1-binding motifs of Kip2 (see main text). Mutation of these motifs does not abrogate but weakens the Kip2-interaction in an additive manner. The Kip2–Bim1 interaction is almost lost upon deletion of the N-terminus (Δ70) or upon mutation of the N-terminal GSK-3 clusters to aspartates (allD, refer also Fig. 1A).

During sequence analysis of the N-terminal extension, we also identified a putative EB1/Bim1-binding (SxIP) motif (Honnappa et al., 2009) at aa 21-24, with S21 being part of the GSK-3 cluster A (SNIP^1^; Fig. 5A; see also Roberts et al., 2014). The site might be conserved in the proposed fission yeast Kip2 homologue Tea2 (Fig. 5A). A second motif could be identified starting at aa 415 (SNIP^2^). The SNIP^1^ Bim1 binding site at aa 21–24 is of particular interest because Bim1 (and also Bik1) act as a processivity factor for Kip2 (Bieling et al., 2007; Roberts et al., 2014). Phosphorylation next to EB1 binding motifs has been shown to disrupt interactions with EB1 (Honnappa et al., 2009; Roberts et al., 2014; Zumbrunn et al., 2001). In this case, the SNIP^1^ motif is flanked by the Mck1 clusters A and B, the first of which includes S21 of SNIP^1^. This suggested that Mck1-dependent phosphorylation interferes with the Kip2–Bim1 interaction. Therefore, Mck1-dependent Kip2 phosphorylation might control plus-end accumulation of Kip2 by regulating Kip2 interaction to its processivity factor(s), in addition to its interaction with MTs.

We, thus, first tested whether the identified motifs are required for Kip2 interaction with Bim1 *in vivo*. Indeed, ^HA^Bim1 co-immunoprecipitated Kip2^13myc^ from cell extracts (Fig. 5B), whereas Bik1 was dispensable for this interaction *in vivo* [Bim1 and Bik1 physically interact with each other (Blake-Hodek et al., 2010; Wolyniak et al., 2006]. The truncated Kip2 variants that lack either the first 37aa (Δ37-Kip2^13myc^, lacking also SNIP^1^) or the entire N-terminal Kip2 extension (Δ70-Kip2^13myc^) almost completely lost interaction with ^HA^Bim1 (Fig. 5B), although the effect was difficult to assess because the cellular levels of these Kip2 variants were also decreased. However, mutation of the N-terminal SNIP^1^ to SNSS abrogated the interaction between Kip2 and Bim1 (Fig. 5C, for localisation of the Kip2-variants see Fig. 5D).

*In vitro*, recombinant ^MBP^Kip2ΔC^GFP^ protein interacted with the EB1 domain of Bim1 (GST-^EB1^Bim1) and this interaction was abrogated after deletion of the N-terminal sequences that included the SxIP^1^ motif (Fig. 5E). Mutation of the N-terminal SxIP^1^ motif reduced, but did not abolish, the interaction with GST-^EB1^Bim1, whereas additional mutation of the second SxIP^2^ motif weakened the interaction further (Fig. 5E). Importantly, deletion of the first 70 N-terminal aa of Kip2 essentially abrogated binding. Hence, we concluded that the N-terminal SNIP^1^ is, indeed, a Bim1-binding site in Kip2.

Does Mck1 regulate the interaction between Kip2 and Bim1? In support of this notion, we found that only the hypo-phosphorylated form of Kip2 co-immunoprecipitated with ^HA^Bim1 *in vivo* (Fig. 5C). Importantly, the recombinant allD-^MBP^Kip2ΔC^GFP^ variant failed to interact with the EB1-binding domain of Bim1 *in vitro* (Fig. 5E). Therefore, these data suggest that the N-terminal Kip2 domain contains a functional EB1-interaction motif that mediates interaction of Kip2 with Bim1, and that phosphorylation of Kip2 by Mck1 close to the EB1-binding motif inhibits the interaction between Kip2 and Bim1. Thus, Mck1-dependent phosphorylation interferes with the cellular activity of Kip2 through two different mechanisms; first, by reducing its overall affinity to MTs and, second, by inhibiting Kip2 binding to its processivity factor Bim1.

### Kip2 phosphorylation controls the amount of Kar9 and dynein on astral MT plus ends

**Fig. 6.**
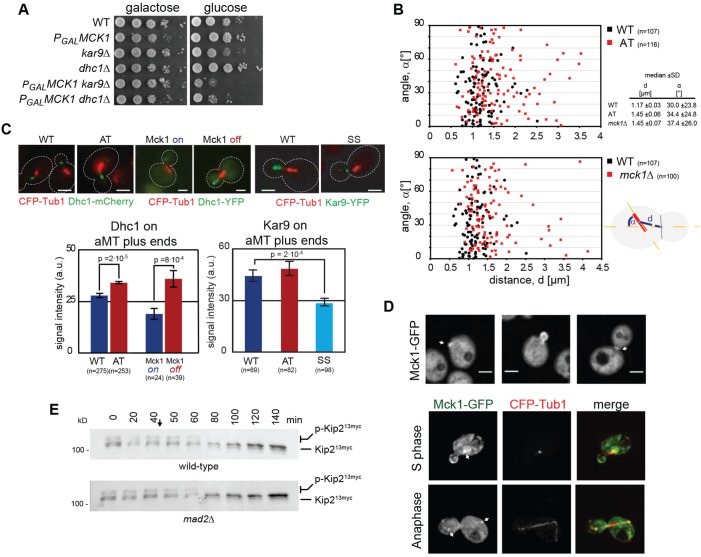
**Kip2 phosphorylation controls the amount of Kar9 and dynein on aMT plus ends and spindle positioning.** (A) Genetic interactions of *MCK1* with genes required for spindle positioning. *P_GAL_^HA^MCK1 kar9*Δ and *P_GAL_^HA^MCK1 dyn1*Δ cells display slow growth upon deactivation of *MCK1* expression on glucose-containing plates. (B) Spindles are mispositioned in *mck1*Δ cells and cells expressing Kip2-AT. Plotted is the angle of the spindle to the mother-bud axis (α, *y*-axis), against the distance from the bud neck (d, *x*-axis), the vertical line denotes the bud neck. Number of spindles counted in brackets. (C) Fluorescence intensity of Dyn1–YFP and Kar9–YFP on aMT plus ends in cells expressing the Kip2 variants indicated or in cells expressing wild-type Kip2 in ^HA^Mck1 overexpressing or depleted cells. Numbers of counted aMTs in brackets, arbitrary intensity units ±s.e.m. are shown. Scale bar: 3 µm. (D) Localisation of Mck1–GFP. A Mck1 sub-pool localises to the incipient bud, to the bud cortex until G2/M phase, and the bud neck. Mck1 also localises to the spindle poles (possibly SPBs or kinetochores) from S phase until anaphase. Scale bar: 2.5 µm. (E) Phosphorylation of Kip2 requires MTs. Time course showing Kip2^13myc^ phosphorylation in extracts of wild type- and spindle assembly checkpoint-defective *mad2*Δ cells, after MT depolymerisation. Nocodazole was added at 40 min (arrow). Equal number of cells were used to generate the extracts for each time point.

We examined the implications of Mck1-dependent Kip2 regulation in spindle positioning. As mentioned, Kip2 is required for deployment of dynein and Kar9 to aMT plus ends (Maekawa et al., 2003; Sheeman et al., 2003). Thus, we first analysed genetic interactions of *MCK1* with the dynein and the Kar9 pathways. Both pathways require aMTs for their function, and their simultaneous genetic deactivation is lethal for yeast cells. Depletion of Mck1 resulted in growth defects of cells deleted for either *DYN1* (the dynein heavy chain) or *KAR9* (Fig. 6A), suggesting that Mck1 is, indeed, required for the correct function of both pathways and consistent with the role of Mck1 in regulation of aMT function by Kip2 phosphorylation. In agreement with this idea, we found that a fraction of spindles were mispositioned in *mck1*Δ cells as well as in cells expressing the hypo-phosphorylated Kip2-AT variant (Fig. 6B) – mainly because of an increase in the distance of the spindle from the cleavage apparatus at the bud neck.

As Kip2 is able to transport dynein to the MT plus end (Roberts et al., 2014), we next examined whether lack of Kip2 phosphorylation by Mck1 would affect deployment of dynein to aMT plus ends. For this, we quantified localisation of dynein on aMT plus ends in cells expressing the hypo-phosphorylated Kip2-AT variant. Dynein accumulation at aMT ends increased in these cells (Fig. 6C). Depletion of Mck1 (which was expressed under the repressible *P_GAL1-10_* promoter) also resulted in increased amounts of dynein at aMT ends, whereas Mck1 overexpression led to the opposite effect (Fig. 6C). Thus, we propose that Mck1 controls plus-end localisation of dynein by adjusting the MT affinity and processivity of Kip2.

The protein Kar9 requires Bim1 for aMT localisation (Miller et al., 2000) and also depends on Kip2 to be transported to the plus ends of aMTs (Carvalho et al., 2004; Maekawa et al., 2003). We, therefore, also examined whether the interaction between Kip2 and Bim1 is required for localisation of Kar9 complexes to MT plus ends. Intensity of Kar9 at aMT plus ends was clearly reduced in cells expressing Kip2-SS as a sole Kip2 source (Fig. 6C). Intriguingly, expression of the hypo-phosphorylated Kip2-AT variant did not increase the amount of Kar9 at bud MT plus ends significantly. However, because Mck1 phosphorylation inhibits binding of Kip2 to Bim1 and the Kip2-SS variant showed reduced targeting of Kar9 to aMT plus ends we, nevertheless, think that Mck1 might control the plus-end deployment of Kar9 as well as dynein.

### Phosphorylation of Kip2 requires MTs and might take place at cortical sites

Mammalian GSK-3 is spatially regulated at cortical sites (Etienne-Manneville and Hall, 2003; Wittmann and Waterman-Storer, 2005). We, thus, asked whether phosphorylation of Kip2 by Mck1 takes place at specific cellular sites, and closer examined the localisation of Mck1 and Rim11 by using spinning disc confocal microscopy and subsequent deconvolution. Mck1–GFP is located throughout the cytoplasm and the nucleus, as previously reported (Huh et al., 2003). In addition, Mck1–GFP accumulates at the cortex of emerging and small buds, at the bud neck, as well as at the spindle poles – either at the spindle pole bodies (SPBs – the yeast MT-organising centres) or at kinetochores (Fig. 6D). Rim11–GFP displays similar localisation but does not localise to the spindle poles (data not shown). The localisation of Mck1 suggests that Kip2 phosphorylation takes place either in the cytoplasm at the cortex or – because Kip2 localises exclusively on aMTs – at the spindle poles.

To further clarify this, we asked whether aMTs are required for Kip2 phosphorylation. After depolymerisation of aMTs with nocodazole, phosphorylation of Kip2 was lost within 20 min (Fig. 6E). Loss of phosphorylation is independent of the nocodazole-induced cell cycle arrest because it still occurred in *mad2*Δ cells. In view of the cortical localisation of Mck1 and Rim11, these data suggest that Mck1 does not phosphorylate Kip2 in the cytoplasm or at the SPB but, possibly, as aMTs reach the cell cortex. It is, therefore, possible that Mck1 controls aMT function by phosphorylating Kip2, thereby regulating dynein-plus-end and Kar9-plus-end turnover at cortical sites (see discussion).

## DISCUSSION

In this work we show that Mck1 phosphorylates Kip2 by regulating aMT length, and the load of dynein and Kar9 complexes at aMT plus ends. We demonstrate that phosphorylation occurs mainly at the entire S/T-rich N-terminal part of Kip2. Phosphorylation of Kip2 by Mck1 occurs in a sequential manner and requires the S63. Mck1 has been shown to prefer substrates that are pre-phosphorylated in close vicinity to the GSK-3 consensus site (Tan et al., 2014), similar to its mammalian counterpart (Fiol et al., 1988). *In vitro*, replacing GSK-3β sites with D or E residues supports the phosphorylation of S/T residues that are located further upstream (Roach, 1991). However, mutation of S63 in Kip2 to D or E failed to mimic and, rather, abrogated phosphorylation (data not shown).

Two lines of evidence suggests that phosphorylation of T275 within the GSK-3 consensus site that is found in the motor domain of Kip2 contributes to Kip2 regulation. First, the double S63A T275A mutation caused the strongest effects regarding aMT stabilisation and resistance of cells to the MT destabilising effect of Benomyl (Fig. 3D). Second, phosphorylation by Mck1 was reduced after introduction of the T275A mutation *in vitro* (Fig. 1F). We, thus, think that phosphorylation of Kip2 at T275 contributes to Kip2 regulation through Mck1, but how exactly T275 phosphorylation affects Kip2 function remains unclear.

It is also not clear which kinases prime Kip2 for phosphorylation downstream of the GSK-3 consensus sites. Our *in vivo* experimental data do not support the *in vitro* data, which suggests that Cdc28 acts as a priming kinase at S63 (Ubersax et al., 2003). In fact, later proteomic studies did not identify Kip2 among the *in vivo* Cdc28 substrates (Holt et al., 2009). In contrast, Dbf2/Dbf20 is a strong candidate for a priming kinase: phosphorylation of Kip2 was reduced in *dbf2-2dbf20*Δ cells and deactivation of Dbf2 kinases led to aMT stabilisation. Moreover, the N-terminal extension of Kip2 contains nine sequences that each match the Dbf2 consensus site. In addition, Kip2 phosphorylation is cell cycle regulated and increases in anaphase – at the time of Dbf2 activation, whereas this regulation is lost upon Dbf2 deactivation. Further experiments are required to address this issue, which is complicated by the fact that other kinases seem to act on the Kip2 N-terminus, mainly at aa positions 69 and 72.

Importantly, lack of Kip2 phosphorylation by Mck1 stabilizes aMTs in a manner that is similar to the overexpression of Kip2 (Carvalho et al., 2004). Therefore, aMT stabilisation in cells with reduced Kip2 phosphorylation could be due to increased amount of Kip2 within the cell. Several lines of evidence argue against this idea: first, cellular steady-state levels of the aMT-stabilising Kip2-AT and Kip2-AA variants did not significantly differ from wild type (for example Fig. 1B or Fig. S1A). Second, cellular Kip2 levels did not increase upon Mck1 downregulation or decrease upon Mck1 overexpression (Fig. 1C). And, third, turnover of Kip2-AA was indistinguishable from wild-type Kip2 (Fig. 4B). In contrast, the stabilising effect of Kip2-AT on aMTs can be adequately explained by the finding that loss of Kip2 phosphorylation increases the amount of the Kip2 that is able to bind aMTs, a prediction that we confirmed *in vivo* (Fig. 4A) and *in vitro* (Fig. 4C). This, in turn, suggests that aMT stabilisation in Kip2-AT-expressing cells is due to increased transport of the Kip2 cargo Bik1 to aMT plus ends. Intriguingly, however, we did not detect any significant increase of Bik1 at aMT plus ends in Kip2-AT-expressing cells, thus the molecular mechanisms that cause aMT stabilisation in these cells remain to be determined.

We found that Kip2 interacts with Bim1 through an EB1-interaction motif that is present inside the Kip2 N-terminal domain, close to the Mck1 and Dbf2 phosphorylation sites. Although we can efficiently detect the Kip2–Bim1 interaction in pull-down assays, the complex is probably not very stable because it cannot withstand gel filtration (Roberts et al., 2014). Furthermore, we propose that a similar N-terminal extension of Tea2 (the fission yeast Kip2 homologue) also contains two EB1-binding sites. The fission yeast EB1 homologue Mal3 binds to the N-terminus of the kinesin-like protein Tea2 acting as a processivity factor, because Tea2 does not efficiently track MTs in the absence of Mal3 (Bieling et al., 2007; Browning and Hackney, 2005). Similarly, Bim1 and Bik1 act as processivity factors for Kip2; they are required for Kip2 in order to counteract the minus-end force when it transports dynein *in vitro* (Roberts et al., 2014). This evidence is consistent with our observation that abrogation of Kip2 phosphorylation leads to Kip2- and dynein accumulation at aMT plus ends, and that the Bim1–Kip2 association is required for efficient transport of Kar9 to the aMT plus ends. However, it is not clear how Kip2 transports Kar9 to the ends of MTs. Acting as a processivity factor for Kip2, Bim1 might indirectly affect Kar9 transport to aMT plus ends (Browning and Hackney, 2005). Alternatively, the Bim1-Kar9 complex might be transported by ‘hitchhiking’ on Kip2, although it is difficult to envisage how Bim1 would bind simultaneously to both proteins. A third possibility is that Kip2 indirectly alters plus-end localisation of Kar9 by affecting deployment of Bim1 to aMT ends.

**Fig. 7.**
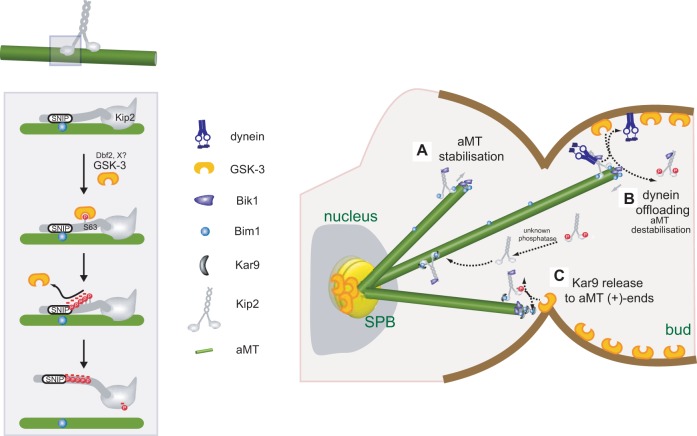
**Model on how Mck1 regulates aMT functions.** Left: Mck1 phosphorylates Kip2 at the N-terminal extension, including the GSK-3 consensus site that comprises S63, close to the EB1-binding motif (SNIP^1^). Phosphorylation disrupts interaction between Kip2 and Bim1. In addition, phosphorylation (involving possibly also T275) causes dissociation of Kip2 from aMTs. Dbf2 and unknown kinase(s) act as priming kinases for Mck1. Right: Mck1 might regulate aMT function through Kip2 phosphorylation in three ways. (A) Unphosphorylated Kip2 binds and stabilises aMTs. (B) When aMTs reach the cortex, Mck1 phosphorylates Kip2, leading to Kip2 dissociation from aMTs and offloading of dynein at cortical sites. The process may be enhanced upon anaphase onset, the time of Dbf2 activation. (C) Phosphorylation by Mck1 also disrupts interaction between Kip2 and Bim1, leading to release of transported Bim1–Kar9 complexes at aMT plus ends. Dephosphorylation of Kip2 by a phosphatase restarts the cycle.

Our data suggest that Kip2 phosphorylation by Mck1 weakens the association of Kip2 with Bim1 and aMTs. In this manner, Mck1 and Dbf2 could control aMT dynamics at the cell cortex (Fig. 7). According to this model, Kip2 becomes phosphorylated by Mck1 as it reaches the cortex. Phosphorylated Kip2 dissociates from aMTs, causing aMT destabilisation (Fig. 7A,B). Dissociation of the Kip2–Bik1 complex from aMTs upon phosphorylation by Mck1/Dbf2 might also be part of the mechanism that is used to offload dynein at the cell cortex (Fig. 7B). This idea is also supported by the finding that overexpression of Mck1 results in reduction of dynein at aMT plus ends, whereas depletion of Mck1 has the opposite effect. By contrast, when Mck1/Dbf2 phosphorylates Kip2, it disrupts not only the interaction between Kip2 and aMTs but also the interaction between Kip2 and Bim1. This would lead to dissociation of Bim1 (and possibly of Kar9) from Kip2, and its deployment to aMT plus ends (Fig. 7C). Our model predicts that both dynein- and Kar9-dependent spindle positioning are defective in cells that lack Mck1, as supported by the genetic interactions of *mck1*Δ with both *dyn1*Δ and *kar9*Δ. *In vitro* assays are certainly required to test these ideas in detail.

To this date, the role of yeast GSK-3 in the control of aMT dynamics was largely unclear. This study fills the gap concerning GSK-3 function between yeast and mammalian cells. Kip2 stabilises aMTs, binds to Bim1 and promotes transport of APC-related Kar9 to aMT plus ends in budding yeast. This resembles the role of kinesin KIF17 – which was recently found to stabilise MTs, interact with EB1 and localise APC to MT plus ends – in mammalian cells (Jaulin and Kreitzer, 2010). We found that Mck1 accumulates at cortical sites of polarised growth in yeast cells, regulates aMT dynamics, and the amount of Kip2 and dynein at aMT plus ends. Moreover, Mck1 regulates the interaction between Kip2 and Bim1. We also showed that the Kip2–Bim1 interaction is required for efficient localisation of Kar9 to aMT plus ends. In mammalian cells, GSK-3 phosphorylates +TIPs, such as CLASP2 or APC, and weakens their interaction with MTs or other +TIPs (Rubinfeld et al., 1996; Zumbrunn et al., 2001), whereas local inhibition of GSK-3 stabilises MTs and promotes their interaction with the cortical cytoskeleton during cell migration (Akhmanova et al., 2001; Etienne-Manneville and Hall, 2003; Kumar et al., 2009; Watanabe et al., 2009). Regulation of Kip2 through Mck1 in budding yeast might be another example for a GSK-3-dependent mechanism to couple the regulation of MT dynamics with the control of aMT-cortical interactions.

## MATERIALS AND METHODS

### Yeast growth and medium

All strains were derivates of S288C (*ura3-52 lys2-801 ade2-101 trp1-Δ63 his1-Δ200 leu2-Δ1*) or BY4743 (for details see Table S1) and grown on standard medium.

### Sequence analysis and generation of Kip2 variants

Sequence analysis to identify phosphorylation sites was performed by NetPhosK (Technical University of Denmark, http://www.cbs.dtu.dk/services/NetPhosK/) and the Eukaryotic Linear Motif resource (Gould et al., 2010) (ELM; http:/elm.eu.org/). All Kip2 variants were generated by site-directed mutagenesis. Untagged versions as well as Venus-YFP-tagged Kip2 variants were cloned into the integrative pRS305 plasmid under control of the endogenous *KIP2* promoter and integrated into the *LEU*2 locus. 13×Myc-tagged (^13myc^) Kip2 variants expressed under control of the *KIP2* promoter were sub-cloned into the pRS314 plasmid. For full plasmid list see Table S2.

### Microscopy

Microscopy was carried out by using an Olympus IX81 microscope equipped with the Cell R software (Olympus Germany, Hamburg). MTs were visualised by integrating a GFP–Tub1- or CFP–Tub1-encoding plasmid into the *URA3*, *LYS2* or *TRP1* locus and analyzed in small-budded cells with spindles no longer than 2 µm by using ImageJ. Length measurements were made using ImageJ. Fluorescence intensities were measured with ImageJ in an area of 0.3 µm (a circle with a radius of 0.32 µm) in the relevant focal plane of a nine-image *z*-stack after background subtraction. Only bud aMTs of metaphase cells were examined. This restriction takes into account the temporal and spatial localisation pattern of Mck1 and Rim11 (see Fig. 6D). Mck1–GFP and Rim11–GFP imaging was performed using a Perkin Elmer spinning disc on a Nikon TE2000 inverted microscope. Deconvolution was performed with Huygens Essential 3.4 (Scientific Volume Imaging, Hilversum, The Netherlands).

### Statistical analysis

Statistical comparison of datasets was made by using a two-tailed *t*-test of unequal variance. The number of measurements was large enough to allow generation of histograms. aMT lengths are stochastic and expected to follow a normal distribution.

### Protein expression and purification

#### His_6_eGFP–Kip2, His_6_eGFP–Kip2-AA, His_6_mCherry–Bik1

All proteins were expressed in SF9 cells from a pFastBacM13 vector integrated in its corresponding Bacmid system carried by DH10Bac cells (BAC-TO-BAC™ expression system; Invitrogen, Paisley, UK) according to Wasilko et al. (2009). Cells were lysed in 50 mM HEPES pH 7.5, 150 mM NaCl, 3 mM EGTA, 1.5 mM MgCl_2_, 0.5 mM ATP, 5% glycerol, 0.1% Tween-20, 15 mM sodium pyrophosphate plus protease inhibitors. Purification was carried out in a two steps by using Blue Sepharose (6 Fast Flow) and Ni^2+^-Sepharose (HisTrap HP, both GE Healthcare Bio-Sciences AB, Uppsala, Sweden).

#### Mck1-CBP

Yeast cells expressing Mck1-TAP expressed from its genomic locus were lysed in 50 mM Tris pH 7.5, 300 mM NaCl, 1.5 mM MgCl, 0.5 mM DTT, 0.1 mM ATP, 10% glycerol, 0.01% NP-40, complete protease inhibitor and 1 mM β-phosphoglycerate. Bait protein was allowed to bind to IgG-Sepharose (6 Fast Flow, GE Healthcare Bio-Sciences AB, Uppsala, Sweden) and bound protein was eluted by incubating with TEV-protease.

#### His_6_Bim1, GST-Kip2^1-80^, GST-^EB1^Bim1, and ^MBP^Kip2ΔC constructs

Cells expressing the corresponding proteins from the pET28 vector in the Rosetta host strain (Merck, Darmstadt, Germany) were lysed in 50 mM Tris pH 7.5, 200 mM NaCl, 1 mM MgCl_2_, 1 mM EGTA, 0.01% NP-40 plus protease inhibitors (Complete™, Boehringer) and purified using standard conditions. After elution, the proteins were dialysed against the lysis buffer, aliquoted and snap-frozen in liquid nitrogen.

### Western blots, cycloheximide-chase, immunoprecipitation and immunostaining, metabolic labelling

#### Western blots

Protein extraction was performed as described in (Knop et al., 1999) or with glass beads in 50 mM Tris pH 7.6, 150 mM NaCl, 1 mM MgCl_2_, protease- and phosphatase-inhibitors and 0.1% NP-40 (better resolution of phosphospecies). For visualisation of Kip2 phosphoisoforms cell lysates were analyzed by SDS-PAGE (6%) and western blotting. Antibodies used for western blots were primary antibodies anti-Myc (1:2000, Rabbit polyclonal, Sigma) anti-HA (1:2000, 12CA5, ABGENT, San Diego, CA), anti-GFP (Rabbit polyclonal, gift from Johannes Lechner, Univ. Heidelberg), anti-Arc1 (1:40,000, Rabbit polyclonal, gift from Ed Hurt, (Univ. Heidelberg), anti-His (1:1000, 6-His, Covance, Emeryville, CA), rabbit PAP, polyclonal (1:1000, DakoCytomation) and anti-tubulin (1:1000, DM1A, Sigma-Aldrich, St Louis, MO), and secondary antibodies anti-mouse IgG peroxidase (Fc-part) and anti-rabbit IgG peroxidase (entire molecule) (both 1:10,000, goat, Sigma-Aldrich). For cycloheximide-chase cells were grown in selective medium, protein translation was stopped by addition of 500 µg/ml cycloheximide and samples were analysed by western blotting. For CIP-treatment lysates were treated with 0.5 u/µg protein CIP or phosphatase inhibitor cocktail II (Sigma-Aldrich). The reaction-mix was incubated for 30 min at 37°C.

#### Immunoprecipitations of HA-Bim1 and HA-Bik1

*P_GAL1_*-dependent expression was induced at OD_0.5_ for 4 h by adding galactose to 2% final concentration. Cells were lysed in 50 mM Tris pH 7.6, 150 mM NaCl, 1 mM MgCl_2_, protease- and phosphatase-inhibitors and 0.1% NP-40 and proteins precipitated overnight with 2 µg anti-haemagglutinin (HA) antibody (clone 7HA, Sigma-Aldrich) and protein-A–Sepharose.

#### Immunostaining

Infected SF9 cells were harvested, washed twice with PBS and fixed with 4% paraformaldehyde. After washing with PBS, permeabilisation with 1% Triton X-100 in PBS for 10 min and additional washing with PBS, cells were blocked in PBS+0.1% Tween+1% BSA for 1 h at 4°C. Cells were stained overnight at 4°C with anti-tubulin antibody (1:250 in blocking buffer, DM1A, Sigma-Aldrich), washed three times for 10 min with PBS and incubated for 1 h at 4°C with secondary antibody (anti-mouse Alexa-Fluor-680, 1:1000 in blocking buffer; Molecular Probes/Invitrogen, Invitrogen, Paisley, UK). Cells were again washed three times with PBS and mounted for microscopy.

#### Metabolic labelling

Cells were grown to an OD_0.5_ in low phosphate YPD; 50 ml were harvested and re-suspended in 1 ml low-phosphate YPD with 4% glucose. Cells were then grown for an additional hour in the presence of 500 µCi/ml inorganic ^32^P phosphate. After cell lysis, Kip2^13myc^ was immunoprecipitated (see above) using anti-Myc (1:2000, Rabbit polyclonal) antibody for 2 h at 4°C and analyzed by western blot.

### *In vitro* kinase assay and *in vitro* binding

The His_6_-tag of eGFP–Kip2 was cleaved off by His_6_-TEV protease. Uncleaved His_6_–eGFP as well as the TEV-protease itself was removed with metal affinity beads (Talon IMAC, Clonetech Laboratories, Mountain View, CA). Six pmol of purified eGFP–Kip2 were incubated with 2.5 pmol purified Mck1-CBP for 30 min at 30°C in kinase-buffer (50 mM Tris pH 7.0, 10 mM MgCl_2_, 0.1 mM EGTA, 1 mM DTT, 100 µM unlabelled ATP, 0.3-0.4 µM ^32^P y-ATP). For the subsequent *in vitro* binding assay, an excess of His_6_mCherry–Bik1 (120 pmol) or His_6_Bim1 (300 pmol) was added and the buffer adjusted to 150 mM NaCl (plus complete protease inhibitor). Proteins were allowed to bind to metal affinity beads for 1 h at 4°C and beads were washed five times with IP-Buffer (see above). Supernatants and beads were analyzed by SDS-Page followed by western blotting or autoradiography.

### MT-sedimentation assay

MT polymerisation of 200 mM tubulin was induced by 5% glycerol and 1 mM GTP for 30 min at 37°C. Tubulin was diluted to 10 mM final concentration in 50 mM Tris pH 7.0, 150 mM KCl supplemented with 10 mM Taxol or 30 µM nocodazole. Polymerised and non-polymerised tubulin was separated by centrifugation at 67,000 ***g*** at 28°C through a 40% glycerol cushion. His_6_eGFP–Kip2 was cleared for 15 min at 67,000 ***g*** and 28°C. 7 pmol Kip2 and ∼40 nmol tubulin were incubated for 20 min at 28°C. Bound Kip2 was again separated by centrifugation through a glycerol cushion. Supernatants and MT-containing pellets were analyzed by SDS-PAGE and western blotting as described above.
